# Investigating How Auditory and Visual Stimuli Promote Recovery After Stress With Potential Applications for Workplace Stress and Burnout: Protocol for a Randomized Trial

**DOI:** 10.3389/fpsyg.2022.897241

**Published:** 2022-06-02

**Authors:** Kunjoon Byun, Sara Aristizabal, Yihan Wu, Aidan F. Mullan, Jeremiah D. Carlin, Colin P. West, Kevin A. Mazurek

**Affiliations:** ^1^Well Living Lab, Rochester, MN, United States; ^2^Delos Living LLC., New York, NY, United States; ^3^Graduate Program in Cognitive Science, University of Minnesota-Twin Cities, Minneapolis, MN, United States; ^4^Department of Quantitative Health Sciences, Mayo Clinic, Rochester, MN, United States; ^5^Division of General Internal Medicine, Department of Medicine, Mayo Clinic, Rochester, MN, United States; ^6^Division of Clinical Trials and Biostatistics, Department of Quantitative Health Sciences, Mayo Clinic, Rochester, MN, United States; ^7^Department of Physiology and Biomedical Engineering, Mayo Clinic, Rochester, MN, United States; ^8^Department of Neuroscience, The Del Monte Institute for Neuroscience, University of Rochester School of Medicine and Dentistry, Rochester, NY, United States

**Keywords:** burnout, heart rate, heart rate variability (HRV), electroencephalography (EEG), relaxation room, trier social stress test (TSST), working memory, workplace stress

## Abstract

**Background:**

Work-related stress is one of the top sources of stress amongst working adults. Relaxation rooms are one organizational strategy being used to reduce workplace stress. Amongst healthcare workers, relaxation rooms have been shown to improve perceived stress levels after 15 min of use. However, few studies have examined physiological and cognitive changes after stress, which may inform why relaxation rooms reduce perceived stress. Understanding the biological mechanisms governing why perceived stress improves when using a relaxation room could lead to more effective strategies to address workplace stress.

**Objective:**

The purpose of this research study is to understand how physiological measures, cognitive performance, and perceived stress change after acute stress and whether certain sensory features of a relaxation room are more effective at promoting recovery from stress.

**Methods:**

80 healthy adults will perform a stress induction task (Trier Social Stress Test, TSST) to evaluate how physiological and cognitive responses after stress are affected by sensory features of a relaxation room. After the stress induction task, participants will recover for 40 min in a MindBreaks™ relaxation room containing auditory and visual stimuli designed to promote relaxation. Participants will be randomized into four cohorts to experience auditory and visual stimuli; auditory stimuli; visual stimuli; or no stimuli in the room. Measures of heart rate and neural activity will be continuously monitored using wearable devices. Participants will perform working memory assessments and rate their perceived stress levels throughout the experiment. These measures will be compared before and after the stress induction task to determine how different sensory stimuli affect the rate at which individuals recover.

**Results:**

Recruitment started in December 2021 and will continue until December 2022 or until enrollment is completed. Final data collection and subsequent analysis are anticipated by December 2022. We expect all trial results will be available by early 2023.

**Discussion:**

Findings will provide data and information about which sensory features of a relaxation room are most effective at promoting recovery after acute stress. This information will be useful in determining how these features might be effective at creating individualized and organizational strategies for mitigating the effects of workplace stress.

## Introduction

According to the 2021 Stress in America™ survey, work-related stress is the top source of stress amongst adult workers (American Psychological Association, 2021). Suboptimal workplace conditions, lack of resources and autonomy, unsupportive interpersonal relationships, and excessive workloads have each been identified as contributors to negative physical and mental health (Danna and Griffin, 1999). Healthcare is one profession that is heavily affected by workplace stress and burnout (Hoff et al., 2017; Dyrbye et al., 2019a,b, 2021; National Academies of Sciences Engineering Medicine, 2019; Shanafelt et al., 2019). Clinician burnout affects quality and safety, patient-clinician relationships, productivity, and clinician turnover (West et al., 2018; National Academies of Sciences Engineering Medicine, 2019). Burnout also costs the U.S. healthcare system in excess of $4.6 billion each year (Han et al., 2019). Across professions, several interventions have been tested to mitigate the effects of burnout and workplace stress at both the individual and organizational level (West et al., 2016; Panagioti et al., 2017). Individual-focused interventions (e.g., meditation, yoga, and focused breathing), and organizational strategies (e.g., institutionally funded counseling sessions, mindfulness-based education courses, and stress management programs) can reduce burnout and workplace stress (Hätinen et al., 2007; Lambert and Corpus, 2021; Rolling et al., 2021; White et al., 2021). In the past few years, relaxation rooms have been used as an organization-level approach to reduce workplace stress and burnout. Relaxation rooms are designed to reduce stress (Ruotsalainen et al., 2015; Sass, 2019; White et al., 2021), however, certain features of the rooms might be more effective at reducing stress for one person than another.

The efficacy of relaxation rooms is often determined based on self-report of stress by the individual (Sass, 2019; Sullivan et al., 2019; Putrino et al., 2020; White et al., 2021). Recently, Putrino et al. (2020) found healthcare workers who used their multisensory, nature-inspired recharge room experienced a 59.6% reduction in self-reported stress levels after a single 15-minute session. However, improvements in perceived stress might not always align with changes in physiological or cognitive responses (Guyon et al., 2020; Loch et al., 2020). Understanding how features of a relaxation room affect physiological responses and cognitive function, in addition to perceived stress levels, could lead to a more individualized approach to reducing workplace stress and burnout.

Wearable devices are one approach to measuring an individual’s physiological responses (Can et al., 2019). Acute stress has been shown to increase heart rate (Kirschbaum et al., 1993) and increase self-report of stress and anxiety (Aristizabal et al., 2021b). Neural activity recorded using electroencephalography (EEG) has been shown to detect changes in spectral power during different epochs of a stress induction task. In a systematic review, Vanhollebeke and colleagues found alpha power has been shown to decrease in the presence of a stressor, which was not found in other frequency bands (Vanhollebeke et al., 2022). In recent years, wearable devices have been developed to allow for mobile EEG recordings to study how neural activity changes in response to stress (Schlink et al., 2017) or to different sensory stimuli (Mazurek et al., 2019). Studies have also shown that measures of cognitive performance are impacted by stress (Vedhara et al., 2000; Shields et al., 2016; Jiang and Rau, 2017). However, few studies have looked at how features of a relaxation room affect physiological measures and cognitive function after stress.

Prior studies have demonstrated naturalistic sensory stimuli are effective at promoting recovery after stress (Yin et al., 2020). Experiencing visual and auditory elements from nature has been shown to reduce stress and improve mood (Shibata and Suzuki, 2004; Fuller et al., 2007; Park et al., 2009; Brown et al., 2013; Hedblom et al., 2019). Physiological benefits in relation to heart rate and neural activity also occur after experiencing naturalistic visual and auditory stimuli (Jo et al., 2019; Yin et al., 2020). Cognitive performance has also been shown to be adversely affected by stress but can be improved when exposed to biophilic environments (Shields et al., 2016; Yin et al., 2018). Taken together, we hypothesize that physiological measures and cognitive performance after a stressful event will return to pre-stress levels faster when experiencing both auditory and visual stimuli of the MindBreaks room compared to only auditory or visual stimuli or neither.

The objective of this study protocol is to determine whether different visual and/or auditory features of a relaxation room affect the recovery of physiological signals, cognitive function, and perceived stress after an experimentally controlled acutely stressful event. Understanding how different sensory features promote recovery after stress could lead to an individual- and organization-level approach for using relaxation rooms to mitigate the effects of workplace stress and burnout.

## Materials and Methods

### Recruitment

**FIGURE 1:**
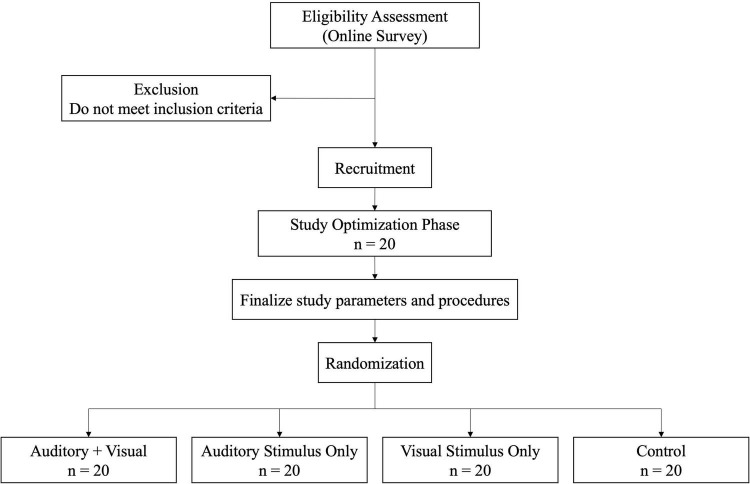
Study design flow diagram.

At the onset of the study, 20 healthy participants above the age of 18 will be recruited during which study parameters will be optimized. Results from these participants will be used to optimize the duration of baseline and recovery epochs of the experiment. After establishing the study parameters, 80 healthy participants will be recruited and randomly assigned into one of four cohorts (20 participants per cohort) as depicted in Figure 1. Cohorts will be randomized and stratified across sex and age, as these factors have been shown to affect physiological responses after stress (Labuschagne et al., 2019; Narvaez Linares et al., 2020). Randomization will be similar to quantitative randomized controlled trials as described by Hong and colleagues (Hong et al., 2018). All participants recruited and consented before the start of the study will be randomized using random assignments generated by a computer to stratify the median age and male/female ratios between cohorts. Any remaining participants recruited after the start of the study will be randomized using block randomization (computer randomized block lengths of 4) to balance the median age and male/female ratios across cohorts consistent with covariate adaptive randomization techniques (Suresh, 2011). Although not directly controlled for in the randomization, we will also ask participants about their job information such as position, length of employment, and perceived workplace stress to be able to characterize with their stress responses during the TSST in exploratory, *post hoc* analyses. Participants mainly will be recruited from a major healthcare organization, however there will be no requirement that participants be healthcare professionals due to the time commitment of the experiment (3 h from approximately 1PM to 4PM).

The inclusion/exclusion criteria for this study are as follows:

Inclusion Criteria:

1. At least age 18
2. Be able to remain in the session for 3-4 h
3. Be able to perform scripted tasks
4. Be able to provide informed consent
5. Be able to wear the wearable devices at all times during the study
6. Have completed a COVID-19 vaccine regimen and can provide written documentation verifying vaccination status at least (14) days prior to the commencement of the research study.

Exclusion criteria:

1. Any reported mood, anxiety, or major health disorders
2. Current use of steroid-based medications
3. History of drug/alcohol abuse
4. Recovering from nicotine dependency and cannot use a nicotine patch for two hours prior to and throughout the study
5. Consumption of excessive amounts of caffeine (more than 400 mg per day as defined by the FDA (Food and Drug Administration, 2018)
6. Severe sleep disturbance based on participants’ self-report (e.g., Sleep apnea)
7. Pregnancy
8. History of cognitive impairment
9. Not completed COVID-19 vaccine regimen
10. Cosmetics or head products that might interfere with the electroencephalography (EEG) recordings.

Eligible participants will be asked to refrain from caffeine two hours before the start of the study due to its effect on the physiological measures of interest. Participants recovering from nicotine dependency will need to use a nicotine patch up to two hours before the study. All consented participants will be screened for symptoms of COVID-19 prior to coming into the lab. Participants and study staff will be required to wear a mask throughout the experiment session. The study protocol has been reviewed and approved by Mayo Clinic Internal Review Board (21-005885).

### MindBreaks Relaxation Room

A MindBreaks relaxation room will be used for this study to determine how the auditory and visual stimuli of the room affect physiological measures, cognitive performance, and self-report of stress during the recovery epoch of the Trier Social Stress Test (see section “Trier Social Stress Test Protocol”). The MindBreaks room is designed to deliver short, effective, and restorative breaks to help increase energy, reduce stress, enhance mood, improve focus, and boost performance (MindBreaks, 2022). The room contains biophilic visual stimuli in the form of an artificial green wall and plants, calming lighting, and a virtual nature skylight to promote relaxation and reduce stress (Berto, 2014; An et al., 2016; Wooller et al., 2018; Hedblom et al., 2019). Immersive natural auditory stimuli from the MindBreaks audio library are played in the room through Sonos^®^ speakers (*Sonos Inc., California, United States*) to provide a calming and relaxing experience (Alvarsson et al., 2010; Largo-Wight et al., 2016; Wooller et al., 2018). For this study, participants will experience the auditory and visual stimuli, auditory stimuli only, visual stimuli only, or no stimuli (control condition), depending on the cohort. Participants who do not experience the biophilic visual stimuli will see neutral curtains covering the walls of the room.

### Trier Social Stress Test Protocol

**FIGURE 2:**
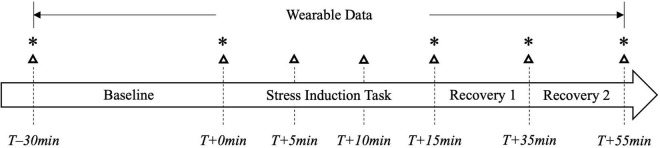
A timeline of the different epochs of the TSST. Dashed lines indicate time points in the experiment when stress-related surveys are administered (indicated by a △) and/or when working memory assessments (SSPAN, OSPAN) are administered (indicated by a *). Wearable devices are recorded continuously from the start of the Baseline epoch to the end of the Recovery 2 epoch. Measures are aligned around the start of the Stress Induction Task (*T* + *0min*).

Participants will perform the Trier Social Stress Test (TSST) to induce an experimentally controlled acutely stressful event (Kirschbaum et al., 1993). The TSST is a widely used laboratory procedure that places participants in a socially evaluative situation that reliably induces mental stress and elicits measurable physiological stress responses (Birkett, 2011). The experimental session is divided into three different epochs: (1) baseline (waiting period upon arrival), (2) stress induction task, and (3) recovery. During the baseline epoch, participants will rest for 30 minutes in the control condition (no biophilic visual or auditory features) to stabilize physiological responses and acquire baseline stress levels. During the stress induction task, participants will perform the TSST which consists of three 5-min phases: (1) the preparation phase, (2) the presentation phase, and (3) the mental arithmetic phase. During the preparation phase, the participant is asked to imagine they have applied for their dream job and given writing materials to make notes about what makes them the ideal candidate. During the presentation phase, the participant will argue why they are the best candidate for the job in front of an interview panel and while being recorded with a video camera and microphone. During the mental arithmetic phase, participants are asked to count backward by 13 starting from 1,022. During the recovery epoch, participants will rest in the MindBreaks room for 40 min while experiencing one of four conditions: auditory-visual stimuli, auditory stimuli, visual stimuli, or control condition (no stimuli). Previous studies have used different durations for each of these epochs (Narvaez Linares et al., 2020). Here, we will adjust the baseline and recovery epoch durations based on how quickly physiological signals and cognitive performance recover during the study optimization period involving 20 participants. Figure 2 depicts a timeline of the different epochs of the TSST, including the times when participants will be asked to perform the cognitive assessment tasks and fill out stress and anxiety self-reports.

### Physiological Measures of Stress

There are numerous physiological responses to stress that can be detected through cardiac and neural measurements (Taelman et al., 2009; Brouwer et al., 2011; Jebelli et al., 2018; Arsalan et al., 2019; Bahadorani et al., 2021). The use and availability of wearable devices for recording such physiological measurements have increased substantially over the last decade (Can et al., 2019). In this study, we propose using multiple wearables to record measures of heart rate (HR), heart rate variability (HRV), and neural activity *via* electroencephalography (EEG). Table 1 describes the different specifications of each of the wearable devices being used in this study.

**TABLE 1:** Features of the wearable devices to acquire physiological signals during a study session.

Device	Sampling rate	Sensor type	Variable measured	On-body location	Data collection application
Apple Watch Series 6	Variable, approximately once every 5-6 s using Workout App	Optical	HR	Wrist non-dominant hand	Apple Health
Empatica E4	1 Hz	Optical	HR	Wrist dominant hand	Empatica E4 Manager
Polar Verity Sense	1 Hz	Optical	HR	Upper arm non-dominant hand	Polar Flow
Polar H10	1 Hz	ECG	HRV	Chest	Elite HRV
Dreem 3	250 Hz	EEG	EEG	Head	Alfin

*Note: HR – Heart Rate, HRV – Heart Rate Variability, ECG – Electrocardiogram, EEG – Electroencephalogram.

#### Cardiac Measures (Heart Rate, Heart Rate Variability)

Heart rate, the number of times the heart beats per minute, will be measured at the following parts of the body: 1) the wrist on the non-dominant hand using an Apple Watch Series 6 (*Apple Inc., California, United States*); 2) the wrist on the dominant hand using an Empatica E4 (*Empatica Inc., Massachusetts, United States*); 3) the upper arm on the non-dominant hand using a Polar Verity Sense (*Polar Electro, United States*); and 4) the chest using a Polar H10 (*Polar Electro, United States*) heart rate monitor. The validity and reliability of HR measurements from Apple Watch Series 6 (Hajj-Boutros et al., 2021), Empatica E4 (Menghini et al., 2019), and Polar Verity Sense (Kim et al., 2021) have been evaluated and are suitable for this research study. Each of these wearable devices will be placed on the participant based on each manufacturers’ guidelines. The Empatica E4 stores data locally on the device, whereas the other devices are paired to an Apple iPhone 11 to record data through the respective applications listed in Table 1.

Heart Rate Variability (HRV), a measure of the variation in time between each heartbeat, is also an important measurement of recovery from stress (Castaldo et al., 2015, 2016; Ghiasi et al., 2020). Here, the Polar H10 (*Polar Electro, United States*) will be used to collect R-R intervals using the Elite HRV app throughout the experimental session and Kubios HRV Premium software (version 3.3.1) will be used to retrieve HRV time- and frequency-domain indices (Lipponen and Tarvainen, 2019). The accuracy of R-R intervals (RR) capturing from Polar H10 is comparable to a reference grade device and suitable for obtaining reliable HRV-related metrics (Umair et al., 2021). Average HR and HRV measurements such as the mean of R-R intervals (Mean RR), a standard deviation of a normal R-peaks (NN) (SDNN), the square root of mean squared difference between normal heartbeats (RMSSD), the percentage of adjacent NN intervals that differ from each other by more than 50 ms (pNN50), very-low-frequency (0.0033 - 0.04 Hz, VLF), low-frequency (0.04 – 0.15 Hz, LF), and high-frequency bands (0.15 – 0.40 Hz, HF) will be collected and analyzed for each epoch of the experiment.

Our selection of HRV metrics is based on a meta-analysis examining HRV in acute mental stress done by Castaldo et al. (2015). Given that time-domain HRV metrics, Mean RR, SDNN, RMSSD, and pNN50 have been shown to be affected by acute stress (Castaldo et al., 2015), we will compare these metrics before, during, after stress to determine when it recovers to baseline levels. Similarly for the frequency domain, HF has been shown to decrease during stress (Castaldo et al., 2015; Shaffer and Ginsberg, 2017), so we will compare the HF of HRV metrics before, during, and after stress to determine when HF power increases back to baseline levels. As exploratory, *post hoc* analyses, we will also look at VLF of HRV to determine if there are any task-related changes after stress. Usui and Nishida (2017) has shown that VLF slowly recovered after a mental task compared to HF, thus it would be worth to examine whether existence of sensory features could promote the recovery process of VLF.

#### Neural Activity

Electroencephalography (EEG) is a well-established, non-invasive approach to measuring neural activity. In this study, the Dreem headband (*Dreem, Paris, France*) will be used to determine how neural activity changes (1) before and after the stress induction task, and (2) when recovering from stress based on the different biophilic features of the recovery room. The Dreem headband has been used to classify sleep stage through EEG and its accuracy is comparable with polysomnography (Arnal et al., 2020; Pépin et al., 2021). Although we are not using the device to study sleep, the neural recordings are expected to be similar in quality during the baseline and the recovery epochs of the study. Additionally, the Dreem headband is a dry electrode system designed for comfort which will be advantageous for the prolonged use during this study. The Dreem 3 headband has 5 electrodes located at O1, O2, F7, F8, and FP2 based on the standard 10-20 orientation (note, FP2 is used as a reference signal). Signals are sampled at 250 Hz and will be recorded using the Alfin app provided by Dreem. To account for the lateralized reference electrode (FP2), we will apply a common average reference to attempt to account for any lateralized effects. EEG signals will be preprocessed to remove artifact noise using independent components analysis. If EEG signals during the speech and mental arithmetic phases are too noisy to analyze, only the preparation phase of the TSST will be included for subsequent analysis.

Electroencephalography analyses will be conducted mainly in python using the MNE toolbox (Gramfort et al., 2013). As the primary outcome, we will compare alpha spectral power (8-13Hz) before, during, and after the stress epoch, as this has been shown to significantly decrease in response to a stressor (Vanhollebeke et al., 2022). As exploratory analyses, we will compare spectral power before, during, and after the stress epoch in the theta (4-8Hz), beta (13-32Hz), and gamma/high-gamma (32-100Hz) frequency bands similar to Arsalan and colleagues (Arsalan et al., 2019), as well as frontal alpha asymmetry similar to prior studies (Quaedflieg et al., 2015; Ehrhardt et al., 2021; Hu et al., 2021). The lateralized reference in the Dreem headband (FP2) will be taken into consideration when interpreting any frontal alpha asymmetry results, as this could produce false positive results. Although these exploratory analyses were not shown to have significant responses to a stressor based on the meta-analysis conducted by Vanhollebeke and colleagues (Vanhollebeke et al., 2022), the focus here will be on whether neural activity in these frequency bands significantly modulates in response to the stressor and the specific sensory stimuli during the recovery epochs, which to our knowledge has not been previously examined.

### Cognitive Assessments

Participants will perform cognitive assessments to assess working memory function at specific times during the stress induction task. Acute stress has been shown to affect performance on working memory tasks (Vedhara et al., 2000; Shields et al., 2016; Jiang and Rau, 2017). In this study, participants will use a cognitive assessment application designed by the Well Living Lab (Jamrozik et al., 2019; Aristizabal et al., 2021a) to perform two working memory tasks: the Operation Span (OSPAN) task and the Symmetry Span (SSPAN) task (Foster et al., 2015; Draheim et al., 2018). Participants will perform these assessments on a 12.9” iPad Pro (*Apple Inc., California, United States*) located on a desk to ensure consistent placement across participants. Although some studies have required 85% accuracy on the processing components of the task (math accuracy for OSPAN, symmetry accuracy for SSPAN), other studies have found similar task performance measures without requiring such a processing accuracy threshold (Đokić et al., 2018). To ensure we have cognitive measures for each time step in the TSST (outlined in Figure 2), we will not require a processing accuracy threshold when analyzing changes in cognitive performance.

Two dependent measures will be used for analysis of both SPAN tasks: Unit Score and Load Score. The Unit Score is the average of the proportion of letters or grids participants can recall correctly in the appropriate order in each set. The Load Score is calculated from the sum of correctly recalled elements from all trials, regardless of the order. Both scores will be used to assess changes in working memory capacity before and after the stress induction task.

### Stress-Related Survey Instruments

In addition to measuring cognitive performance and physiological changes, self-reports of stress will be measured throughout the experiment. To characterize the baseline level of stress, the State-Trait Anxiety Inventory (STAI) (Spielberger et al., 1983) and the Perceived Stress Scale (PSS-10) (Cohen et al., 1983) will be administered before the experimental session. Throughout the experiment, participants will answer a single-question ecological momentary assessment of perceived stress (Likert scale 1-7) and the State component of the short-form of the State-Trait Anxiety Inventory (STAI-6) (Marteau and Bekker, 1992) to dynamically rate their stress levels. These survey responses allow for characterizing changes in the participant’s perceived stress levels compared to the cognitive performance and physiological responses. As an exploratory analysis, surveys regarding job satisfaction (Russell et al., 2004) and the Ten Item Personality Inventory (TIPI) (Gosling et al., 2003) will be distributed before each experimental session to examine how physiological signals during the stress task are related to participants’ general personalities.

### Statistical Analyses

#### Planned Statistical Analyses

Summary statistics of demographics (Age, Sex, Ethnicity, Education) and baseline characteristics (Trait level of Anxiety and Stress) of participants will be reported for all participants in the study (separated by the main and the optimization period). Cognitive performance metrics and survey responses will be analyzed using univariate mixed-effects models. Descriptive statistics, generalized estimating equations (GEEs), and power spectrum analysis will be used to analyze the wearable-collected physiological data. Generalized estimating equations will also be used to fit each of the physiological measures (HR, HRV, and EEG) and compare across the different epochs of the experiment. As an additional analysis, we will use univariate mixed-effects models to understand how physiological, subjective and cognitive measures correlate and differentiate the different cohorts, providing an overall view on how these features combine to represent the recovery after stress.

#### Power Analyses

Sample size was determined based on the 5 cognitive performance measures, which were the least frequent measure collected during each experimental session, with an assumption of using a univariate mixed effects model with 5 measurements per participant, significance level of 0.05, moderate correlation between repeated measures (0.3), and directly comparing two experimental conditions instead of all conditions at once. Powering based on the least frequent measure ensured that we were sufficiently powered when analyzing the other measures in the study. We would reach 80% power with 18 participants per group with a mean difference of 1 standard deviation. Including 20 participants per group will provide a buffer for potential drop-out.

## Discussion

We anticipate the findings from this study will have a wide impact ranging from the scientific community to institutions looking for effective organizational strategies for mitigating workplace stress and burnout. The study optimization from 20 participants is being conducted between December 2021 and March 2022 to test and finalize the study parameters and procedures. Recruitment for the main trial (*n* = *80*) is expected to start in March 2022 and will continue until December 2022 or until enrollment is completed. Participants will be randomly assigned to one of four cohorts and data are collected throughout this period. Final data collection and subsequent analysis are anticipated by December 2022. We expect all trial results will be available by early 2023.

A socially evaluating situation can be stressful for the most individuals. The TSST is a powerful technique for eliciting a stress response in an experimentally controlled environment. However, there are other factors that might contribute to stress responses in a real working environment that might not be captured in the TSST. There are several factors that play a role in workplace stress, including working environment and workload, the role of the employee (and any ambiguity in that role), or the organizational climate (Colligan and Higgins, 2006). An individual’s job function, such as working in a managerial position or in public relations, might also have differing levels of workplace stress that might not be fully captured by the TSST. Even with this potential limitation, the results from this study can provide valuable insights how individuals recover from acutely stressful situations. Understanding how relaxation rooms mitigate responses after acute stress could provide insight into which physiological and cognitive measures to monitor when attempting to detect and mitigate chronic workplace stress and burnout in real workplace environments.

Other potential pitfalls or unintended effects may come from each participants working position which has been shown to have differing levels of stress (Colligan and Higgins, 2006). Additionally, Xin and colleagues have shown that personality index also correlates with response after acute stress (Xin et al., 2017) which could also play a factor in how each cohort responds. Although we cannot statistically power for each of these factors, we will record information about each of them from each participant to examine in *post hoc* exploratory analyses.

Performing the cognitive tasks might induce additional stress by itself, however, we will attempt to characterize this stress due to the cognitive task by analyzing the physiological measures during the cognitive task at baseline and at the end of the recovery epoch. Additionally, we are assuming that the findings from this study can be extended to understand how longitudinal use of a relaxation room might mitigate the adverse effects of burnout and workplace stress. Future studies are needed to test these assumptions as the physiological and cognitive responses after acute stress might differ from chronic stress.

The strength of this proposed study is the ability to examine how effective different sensory features of a relaxation room are at promoting recovery of physiological signals, cognitive function, and perceived stress after a highly controlled stress induction task. Using multiple wearable devices to acquire various types of physiological signals including heart rate and neural activity as well as using two different working memory tasks to characterize cognitive performance allows us to acquire high-resolution information about how stress influences each individual. These findings could ultimately lead to improved individualized and organizational approaches to mitigating workplace stress and burnout.

## Ethics Statement

This study involving human participants was reviewed and approved by The Mayo Clinic Institutional Review Board in September 2021, under the number 21-005885. The patients/participants provided their written informed consent to participate in this study.

## Author Contributions

JC, YW, KB, and KM worked on the design of the cognitive assessments. JC implemented the technical framework for cognitive assessments. KB, KM, and SA designed the experimental framework with assistance from CW. AM provided the statistical power analysis for the study. KB and KM wrote the first draft of the manuscript. All authors contributed to writing and revising the manuscript and approved the submitted version.

## Conflict of Interest

This work is supported by Delos Living LLC. This research has been reviewed by the Mayo Clinic Conflict of Interest Review Board and is being conducted in compliance with Mayo Clinic Conflict of Interest policies. Both the Mayo Clinic Conflict of Interest Review Board and the Institutional Review Board have reviewed the Financial Conflict of Interest for KB, SA, YW, JC, and KM related to this research and they have determined that this Financial Conflict of Interest poses no additional significant risk to the welfare of participants in this research project or to the integrity of the research. In alignment with the Financial Conflict of Interest management plan outlined by the Mayo Clinic Conflict of Interest Review Board, the research results for this study will be corroborated by a non-conflicted, non-subordinate staff member with appropriate expertise. The remaining authors declare that the research was conducted in the absence of any commercial or financial relationships that could be construed as a potential conflict of interest.

## Publisher’s Note

All claims expressed in this article are solely those of the authors and do not necessarily represent those of their affiliated organizations, or those of the publisher, the editors and the reviewers. Any product that may be evaluated in this article, or claim that may be made by its manufacturer, is not guaranteed or endorsed by the publisher.
